# Gene expression of *Vibrio parahaemolyticus* growing in laboratory isolation conditions compared to those common in its natural ocean environment

**DOI:** 10.1186/s12866-017-1030-6

**Published:** 2017-05-19

**Authors:** Katherine García, Cristian Yáñez, Nicolás Plaza, Francisca Peña, Pedro Sepúlveda, Diliana Pérez-Reytor, Romilio T. Espejo

**Affiliations:** 1 Centro de Investigación Biomédica, Facultad de Ciencias de la Salud, Instituto de Ciencias Biomédicas, Universidad Autónoma de Chile, Av. El Llano Subercaseaux, 2801 Santiago, Chile; 2 Institute of Nutrition and Food Technology, Universidad de Chile, Av. El Líbano 5524, Macul, Santiago, Chile; 3 School of Bioinformatics Engineering, University of Talca, Talca, Chile

**Keywords:** RNA seq, *Vibrio parahaemolyticus*, Genome, Bioinformatics, sRNA, mRNA

## Abstract

**Background:**

*Vibrio parahaemolyticus* is an autochthonous marine bacterial species comprising strains able to grow in broth containing bile salts at 37 °C, a condition seldom found in the ocean. However, this condition is used for isolation in the laboratory because it is considered a necessary property for pathogenesis. In this context, revealing how gene expression enables *V. parahaemolyticus* to adapt to this particular condition –common to almost all *V. parahaemolyticus* isolates- will improve our understanding of the biology of this important pathogen. To determine the genes of *V. parahaemolyticus* differentially expressed when growing in isolation condition (37 °C, 0.9% NaCl, and 0.04% bile salts) referred to those at the temperature and salt concentration prevailing in ocean south of Chile (marine-like condition; 12 °C, 3% NaCl, and absence of bile salts) we used high-throughput sequencing of RNA.

**Results:**

Our results showed that in the isolation condition, among the 5034 genes annotated in the *V. parahaemolyticus* RIMD2210633 genome, 344 were upregulated and 433 downregulated referred to the marine-like condition, managing an adjusted *P*-value (Padj) < E^−5^. Between the 50 more highly expressed genes, among the small RNAs (sRNA), the three carbon storage regulators B (CsrB) were up four to six times, while RyhB, related to iron metabolism besides motility control, was down about eight times. Among proteins, BfdA, a hemolysin-co-regulated protein (Hcp1) secreted by T6SS1, one of the most highly expressed genes, was about 140 times downregulated in isolation condition. The highest changes in relative expression were found among neighboring genes coding for proteins related to respiration, which were about 40 times upregulated.

**Conclusions:**

When *V. parahaemolyticus* is grown in conditions used for laboratory isolation 777 genes are up- or downregulated referred to conditions prevailing in the marine-like condition; the most significantly overrepresented categories among upregulated processes were those related to transport and localization, while secretion and pathogenesis were overrepresented among downregulated genes. Genes with the highest differential expression included the sRNAs CsrB and RhyB and the mRNAs related with secretion, nutritional upshift, respiration and rapid growing.

**Electronic supplementary material:**

The online version of this article (doi:10.1186/s12866-017-1030-6) contains supplementary material, which is available to authorized users.

## Background

*Vibrio parahaemolyticus* is an autochthonous ocean-dwelling bacterial species comprising strains that are widely disseminated in marine environments throughout the world (see by example review [1]). Some of these strains can cause severe diarrhea when present in seafood [1]. Since traditional isolation of *V. parahaemolyticus* is performed in broth containing bile salts at 37 °C (Bacteriological Analytical Manual of the US Food and Drug Administration [2]) almost all the known strains are able to grow in this condition though it is seldom found in the ocean. Insights into how regulation of gene expression enables *V. parahaemolyticus* to adapt from marine environment to laboratory isolation conditions will improve our understanding of the biology of this important pathogen. We studied this growth adaptation by determining the differential gene expression when growing at isolation (37 °C, 0.9% NaCl and 0.04% bile salt) versus marine-like condition (12 °C and 3% NaCl without bile salt). We used 12 °C and 3% NaCl for the second condition because these are the average superficial seawater temperature and salt concentration in the southern Chile littoral where the arrival of the pandemic strain of *V. parahaemolyticus* caused one of the world’s largest outbreaks of seafood-related diarrhea [3, 4], while we used 37 °C, 0.9% NaCl and 0.04% bile salt because these are the isolation conditions. Differential gene expression upon change in growth temperature, salt concentration and addition of bile salts originally focused on selected genes and lately, with the advent of high-throughput RNA sequencing, on the whole genome. Several studies of gene expression has been compared in different conditions but not between those employed in this study. One of them explore the expression of three selected proteins, superoxide dismutase (SOD), catalase (CAT) and TDH by *V. parahaemolyticus* as influenced by heat shock. They showed that SOD and CAT activities are reduced but TDH protein synthesis is enhanced after heat shock at 42 °C [5]. Other studies have focused on secretion systems. They have received particular attention because of their involvement in pathogenesis. It was shown that Hcp2 of type VI secretion system chromosome II (T6SS2) but not Hcp1 of type VI secretion system chromosome I (T6SS1) is expressed at 37 °C [6]. On the other hand, Livny et al. [6] compared the transcriptomes of *V. parahaemolyticus* cultured under standard laboratory conditions in the presence of bile, and directly isolated from the ceca of infected infant rabbits, which mimic the human infection. They showed that expression of genes encoding type III secretion system chromosome II (T3SS2) and its effectors is markedly upregulated under bile and infection conditions. However, the expression of 277 genes was only altered under infection, suggesting that *V. parahaemolyticus* gene expression during infection is subject to significant regulatory influences in addition to bile induction. More recently, Urmersbach et al. [7] showed that 638 genes are differentially expressed between 15 and 37 °C but virulence-associated genes (*tdh1*, *tdh2*, *toxR*, *toxS*, *vopC*, T6SS1, T6SS2) remained mostly unaffected by temperature.

In this work, we try to understand which genes are involved in the ability of *V. parahaemolyticus* strains to grow in a condition commonly used for isolation in the laboratory, but seldom found in the marine environment. We show that about 15% (777 of 5034) of the genes are differentially expressed under the condition of isolation in the laboratory with respect those expected to prevail in the marine-like condition. We interpret and discuss the overall differences between growth in laboratory isolation and marine-like conditions and discuss in further detail some results that seemed most interesting, namely: Increased expression of the three carbon storage related sRNAs, downregulation of RyhB sRNA which translate into upregulation of genes involved in motility, downregulation of T6SS in chromosome I and II but differential regulation of the T3SS in chromosome I and II, and rise of cold shock proteins, CspA and VPA0552 at 37 °C.

## Methods

### Strains and culture conditions

*Vibrio parahaemolyticus* RIMD2210633, a strain maintained in our laboratory since 2002, was used in all experiments. The strain was grown in two conditions; Marine-like condition (E): Luria-Bertani (LB) with NaCl 3%, 12 °C and Isolation condition (I): LB with NaCl 0.9%, 37 °C, supplemented with bile salt 0.04% (Bile salt for microbiology, Sigma-Aldrich, MO, USA). LB was prepared using yeast extract 0.5% and Tryptone 1%, both from Becton Dickinson (NJ, USA). Preliminary growth studies showed that the generation times of *V. parahaemolyticus* when growing in conditions E and I were 12 and 0.5 h, respectively. Stationary phase was reached at OD_600_ of 4 and 5, respectively.

### Total RNA isolation

RNA was isolated from three parallel independent cultures for both conditions. One and a half ml cultures were harvested in exponential phase at an OD_600_ of 1.0 and rinsed once with PBS. Total RNA was isolated from the pellet with Trizol Max Bacterial (ThermoFisher Scientific) according to the manufacturer’s protocol. The quantity of total RNA was determined by Nanodrop 2000 (Thermo Scientific, Wilmington, DE, USA) and QuantiFluor-ST Fluorometer-Ribogreen (Promega, USA), and quality and integrity of the RNA was determined by electrophoresis in the Bioanalyzer 2100 (Agilent Technologies, USA). Yield of RNA per optical density was 0.6-times lower in condition E than condition I. RNA integrity (RIN) was higher than 7.8 in all RNA samples. No differences in the electrophoretic patterns of the RNA in the two conditions were evident.

### RNA library preparation and nucleotide sequencing

Four micrograms of total RNA was initially depleted of ribosomal RNA using the Ribo-Zero rRNA Removal Kit (Gram-Positive Bacteria, Illumina; catalog no. MRZGP126) according to the manufacturer’s protocol, using Riboguard and purifying the RNA by precipitation with ethanol in the last step. Libraries were then prepared following the TruSeq Stranded mRNA Sample Preparation Guide (Part no. 15031047, Rev. E October 2013), following the low sample protocol (LS) but bypassing the mRNA purification. The following barcodes were used: sample I1, AR013 - AGTCAA(C); sample I2, AR014 - AGTTCC(G); sample I3, AR015 - ATGTCA(G); sample E1, AR016 - CCGTCC(C); sample E2, AR018 - GTCCGC(A); sample E3, AR019 - GTGAAA(C). RNA dissolved in re-suspension buffer was immediately used for first strand cDNA synthesis (beginning at step 12, page 20 of the protocol), with the following options: no control was used and re-suspension buffer was used instead in every occasion indicating addition of control. The clean-up PCR step (page 40) was performed using MagJET NGS Cleanup and Size Selection Kit (catalog no. K2821, Thermo Scientific) instead of the AMPure XP Beads. Libraries were validated using Bioanalyzer High Sensitivity Chip.

Created libraries were sequenced at Beijing Genomics Institute (BGI) on the Hiseq 2000 platform (Illumina, San Diego, USA) using single-end 50 bp, and 19.7 to 25.4 million reads per sample were received from BGI after processing and filtration. The number of reads was reduced by random selection to 19 million reads in each sample using ShortRead Bioconductor package [8]. Subsequent filtration by removing adaptors and quality control of reads reduced the number of reads to 17.4–17.5 million per sample using BBDuk (sourceforge.net/projects/bbmap). Reads of each sample were aligned against chromosome I and II of *V. parahaemolyticus* RIMD2210633 with Burrows-Wheeler Aligner (BWA) [9] using the algorithm BWA-BackTrack. Alignments were ordered by position in the reference genome using SAMtools [10]. Annotation of the *V. parahaemolyticus* RIMD2210633 genome was done using information in RefSeq [11] for open reading frames (ORF), tRNA, and rRNA, and BSRD [12] for sRNA with a custom script. Reads counting for each feature were determined using HTSeq-count with the following parameters: -f bam, −r pos, −s reverse, −a 10, −m union [13]. The overall fragments coverage of genomic regions corresponding to features, such as ORFs, tRNAs, rRNAs, sRNAs, and intergenes were calculated according to the counts obtained and mapped in total alignment reads. Coefficient of Determination (R^2^) was calculated using RPKM (*Reads Per Kilobase of transcripted gene per Million mapped reads with features*), calculated for the counts of the features of all samples using a custom script. The differential expression analysis was performed using the DESeq2 package of Bioconductor [14]. The parameters used for analysis were 3-fold change (log_2_FC: 1.58), Padj 1E^−5^, and the 12 °C growth condition as reference. Gene Ontology (GO) [15] terms were obtained from UniprotKB databases [16].

Clustering of GO terms for the differentially expressed genes was carried out with clusterProfiler Bioconductor [17] using R: A library was created using the locus tag and Entrez ID in the annotated genome described above and the GO terms id associated with each protein, using the *AnnotationForge* Bioconductor package (*AnnotationDbi: Annotation Database Interface*, R package version 1.36.0. [https://bioconductor.org/packages/release/bioc/html/AnnotationForge.html]. Functional partners of the highly expressed genes were predicted using the STRING program [18] to identify networks involved in biological interactions. Overrepresentation of biological functions among up- or downregulated genes was calculated using EnrichGO of clusterProfiler using the same library created above in R, and Padj = 0.01 -calculated using Benjamini & Hochberg method- and q-value = 0.05.

## Results

The natural habitat of pathogenic *V. parahaemolyticus* is seawater, which in southern Chile has average surface water temperature around 12 °C. However, almost every strain of the species is able to grow at 37 °C in bile salt. To compare gene expression between these two conditions RNA was single-end sequenced and raw reads were randomly selected and filtered to approximately 17 million reads for informatics analysis.

### Total transcript abundance

**Fig. 1:**
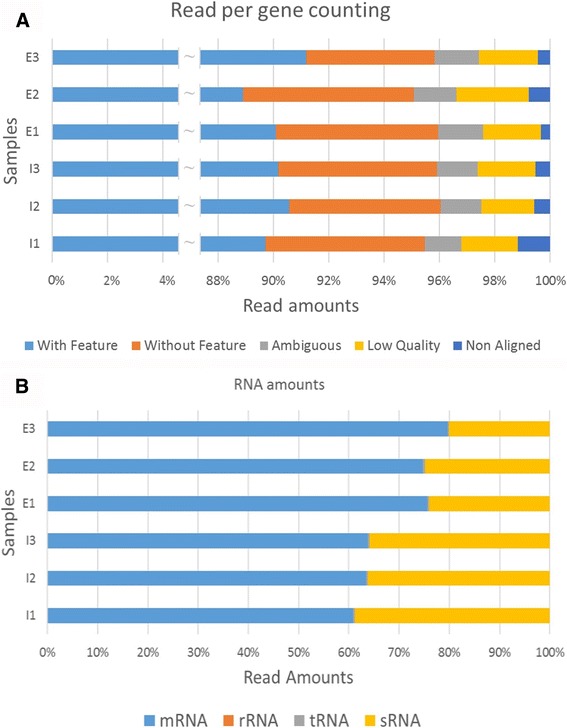
Reads alignment against the annotated genome calculated with HTSeq-count using the following parameters: -f bam, −r pos, −s reverse, −a 10, −m union. E, Marine-like conditions. I, Isolation conditions. **a** Percentage of reads aligned with features and without features and reads not aligned because of lack of similarity. **b** Percentage of reads aligned with different gene classes

Figure 1a shows the results of the counting of the reads aligned against the annotated genome, calculated with HTSeq-count. “With Feature” corresponds to reads aligned to annotated regions, which in this case includes the 43 sRNA described for *V. parahaemolyticus* [12]*.* ‘Without Feature’ corresponds to reads aligned to non-annotated regions and may include sRNA not yet described in *V. parahaemolyticus*, antisense in ORF, UTRs, etc. The percentage of reads corresponding to annotated genes (“With Feature”) and to non-annotated intergenic regions (“Without Feature”) roughly parallels the corresponding percentages of annotated genome; 88.2% annotated and 11.8% non-annotated. Figure 1b shows the percentage of reads aligned to regions with different features: the vast majority of the reads corresponded to mRNA, the sense strands of annotated ORFs, and second in abundance are reads aligning with regions annotated as sRNA. The percentage of sRNA reads observed is more than 200 times the percentage of the genome annotated as such, which covers only 0.12% of the whole genome. This is because some sRNA are present in very large amounts.

### Relative gene expression

The Coefficient of Determination (R^2^) of normalized expression per gene, RPKM was between 0.985 and 0.992 for I samples, 0.952 and 0.974 for E samples, and 0.689 and 0.783 between I and E samples. RPKM values oscillated between 0 and 55,000 for mRNA, and 0 and 400,000 for sRNA. To define the transcriptome (total genes with reads with features) expressed in each condition, we selected genes with RPKM >5 among the 5034 annotated loci. This selection included 3841 genes in I samples and 4143 genes in E samples. This implies that 76% and 82% of the total annotated genes in *V. parahaemolyticus* were significantly expressed in each condition or that 302 gene expressed in E samples were repressed in I samples (Additional file 1).

### Differential expression

To identify genes that were differentially expressed in cultures growing at 37 °C, 0.9% NaCl and 0.04% bile salts versus those growing at 12 °C with 3% NaCl, we compared the RNA-Seq data for these conditions using DESeq2, a differential expression analysis package for RNAseq data that presumes read abundance can be modeled by a negative binomial distribution [20]. Of the 5034 annotated genes in our *V. parahaemolyticus* genome file (Additional file 1), 777 genes in total were significantly affected, 344 (6.8%) and 433 (8.6%) were significantly up- or downregulated (>3-fold, Padj <E^−5^), respectively. Nine of 43 sRNA (21%) were up- or downregulated. Of the 777 genes differentially expressed, 456 genes were from chromosome I and 321 from chromosome II, approximately the ratio of the chromosomes sizes.

### Regulation of the highly expressed genes

Table 1 shows the RPKM, differential expression parameters, and some properties for the 21 genes differentially expressed among the 50 more highly expressed genes (RPKM) in each condition. Five sRNA were among these 21 genes. The three carbon-storage regulator (CsrB) sRNA described in *V. parahaemolyticus* were upregulated between four to six times. CsrB antagonize the activity of CsrA, an RNA-binding protein which coordinates a wide range of cellular physiology including regulation of carbon metabolism, motility, biofilm formation, production of secondary metabolites and quorum sensing, thus affecting virulence [19]. The small 6S RNA was also upregulated about five times; this RNA binds to the σ70-holoenzyme form of RNA polymerase reducing its activity in stationary phase and hence repressing the expression of σ 70-dependent genes [20]. Small RNA svpa113.1, homologous to RyhB was downregulated about eight times; RyhB is expressed when cellular iron concentration is low [21]but also modulates the expression of several genes that control motility, chemotaxis and biofilm formation [22]. Among the highly expressed genes stands up the one encoding BfdA, a hemolysin-co-regulated protein (Hcp1) secreted by T6SS1 and involved in the structural tube formation [23, 24], which is downregulated more than 140 times Also, all the BfdA functional partners genes predicted by the STRING program [18]; VgrG protein, VP1401, VP1402, VP1403, VP1405, VP1406, IcmF-like protein, VP1409, VPA1027 (Hcp2) and VPA1034 were downregulated.

**Table 1:** Differentially expressed genes among the 50 more highly expressed in both growth conditions

Gene	RPKM I	RPKM E	log_2_FC	*p*-value	Observations
Upregulated genes in isolation condition					
svpa172.1	157,710	37,082	2.7	7.2E^−31^	Carbon storage regulator (CsrB)
svpa2439.1	121,815	43,795	2.1	2.2E^−51^	CsrB
svpa3216.1	128,785	50,025	1.9	7.3E^−20^	CsrB
svpa2734.1	429,216	166,980	2.3	1.6E^−25^	6S RNA
VPt061	4867	1639	2.1	2.0E^−16^	Arginine tRNA
VPt108	7708	2916	2.0	6.3E^−28^	Ileucine tRNA
VPA1289	20,530	1102	4.8	4.8E^−151^	CspA
VPA1476	4431	277	4.6	5.4E^−106^	Hypothetical protein
VPA1475	3828	531	3.4	2.3E^−46^	Purine nucleoside phosphorylase DeoD-type 2
VP0129	4086	613	3.3	2.0E^−68^	Phosphoenolpyruvate carboxykinase
VPA0552	55,155	9280	3.1	1.7E^−85^	Cold shock DNA-binding domain protein
VP2585	37,016	9182	2.6	2.2E^−53^	Hypothetical protein
VP0256	4022	1260	2.2	1.4E^−40^	30S ribosomal protein S10
VPA0466	7349	2418	2.2	8.1E^−71^	Universal stress protein A (Usp)
VPA1428	3936	1310	2.2	6.6E^−42^	Azurin: blue copper protein
VP0076	5271	2400	1.7	7.6E^−19^	Usp
VPA1469	22,981	10,708	1.7	3.4E^−26^	Outer membrane protein (OMP)
Downregulated in isolation condition					
svpa113.1	2014	24,669	-3.0	2.7E^−66^	RyhB
VP1393	19	4134	-7.2	0	BfdA, Hcp1
VP0795	1150	11,616	-2.7	1.2E^−116^	Histidine-containing phosphocarrier protein
VP2157	1169	8519	-2.3	2.6E^−27^	Glyceraldehyde-3-phosphate dehydrogenase

Oddly, the cold shock protein CspA is upregulated about 30 times at 37 °C, as well as other cold shock proteins (VPA0552). However, as cited in Discussion section, CspA can also function as a nutritional upshift stress protein [25].

### Genes with the highest differential expression

Table 2 shows the RPKM and differential expression parameters for the 10 genes with highest upregulation and the 10 genes with greatest downregulation. Five neighboring genes VP1512-VP1516, probably all related to respiration, are among those showing the highest upregulation, close to 70 times. Two other neighboring genes (VP1634, VP1635) related to polyamine production are also highly upregulated. Among the most downregulated genes (about 140 times) is the gene encoding BfdA, the hemolysin-co-regulated protein (Hcp1) secreted by T6SS1, found also among the most highly expressed genes. Further, four genes close together in the genome (VP1777, VP1783, VP1794 and VP1796) and three (VP1067, VP1068 and VP1072) are more than 100 times downregulated; they are described as hypothetical proteins but might have related functions.

**Table 2:** RPKM and differential expression parameters for the 25 genes with the highest differential expression between both conditions

Gene	RPKM I	RPKM E	log_2_FC	*p*-value	Observations
Genes upregulated in isolation condition					
VP1512	1104	29	5.7	5.0E^−83^	Hypothetical protein
VP1513	392	11	5.6	1.1E^−89^	Formate dehydrogenase large subunit
VP1514	357	10	5.5	1.9E^−79^	Formate dehydrogenase, iron-sulfur subunit. Interaction with 4Fe-4S
VP1515	216	5	5.9	1.6E^-^^141^	Formate dehydrogenase, cytochrome b556 subunit. Membrane protein
VP1516	671	16	5.9	8.2E^-^^219^	Hypothetical protein
VPA1634	2140	8	8.5	0	Putrescine transporter
VPA1635	3652	16	8.4	0	Ornithine decarboxylase, initial enzyme in polyamine synthesis
VPA0040	679	9	6.7	1.5E^-^^289^	Hypothetical protein
VP0061	330	7	6.0	4.1E^-^^130^	Multidrug transmembrane resistance signal peptide protein
VPA0475	1764	5	8.8	6.1E^-^^228^	Hypothetical protein
Genes downregulated in isolation condition					
VP1393	19	4134	−7.2	0	BfdA, Hcp1
VP1777	0	14	-7.1	4.1E^−24^	Aldehyde dehydrogenase Oxidation of acetaldehyde NAD or NADP reduction
VP1783	0	51	-7.7	3.1E^−29^	Hypothetical protein
VP1794	0	29	-7.5	6.6E^−23^	Hypothetical protein
VP1796	0	21	-7.2	4.7E^−25^	Hypothetical protein
VP1067	0	10	-7.1	6.6E^−20^	Hypothetical protein
VP1068	1	178	-7.0	9.0E^-^^114^	Hypothetical protein
VP1400	1	207	-7.5	3.5E^-^^248^	Hypothetical protein
VP1072	1	142	-7.2	1.1E^-^^186^	Helicase
VPA1424	2	492	−7.2	0	Fructose transport

### Overall differential expression

**Fig. 2:**
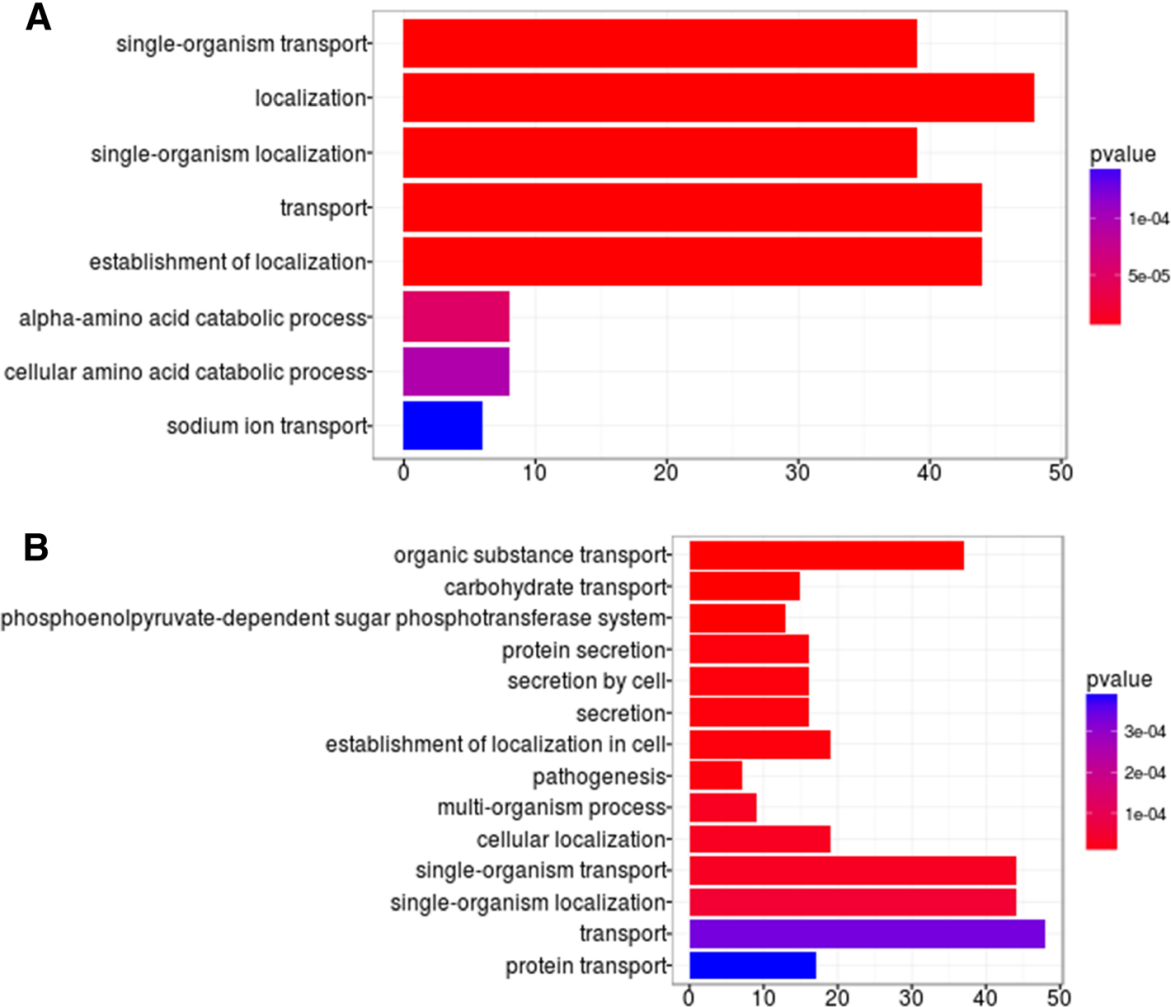
Analysis by gene ontology terms. Most significant overrepresentation for genes in different biological functions that were (**a**) upregulated or (**b**) downregulated in the isolation condition

To compare the expression of all the differentially expressed genes, they were grouped by gene ontology term using enrichGO from clusterProfiler [17]. An overrepresentation analysis of gene ontology terms was performed for up- and downregulated genes and the results are shown in Fig. 2a and b, respectively. Among the 777 differentially expressed genes, 753 were associated with a GO term. Small RNAs do not have GO terms and hence they are not included in this particular comparison. The most significantly overrepresented upregulated processes were those related to transport and localization. Among overrepresented processes with downregulated genes were secretion- and pathogenesis-related genes.

## Discussion

Expression of 777 genes differed when the RNA of *V. parahaemolyticus* grown at 37 °C, 0.9% NaCl in the presence of bile salts (conditions for laboratory isolation) was compared with the same grown at marine like-conditions with temperature 12 °C and salt 3% NaCl, (more common in the marine environment). Expression of genes measured as RPKM varied from zero up to hundreds of thousands while the extent of change varied from 3 up to 200 times. Some of the more interesting changes when comparing the bacteria growing in isolation versus marine like conditions were:

- Increase in expression of the three CsrB sRNAs genes probably related to the faster carbon metabolism required for growing 24 times more rapid at 37C.
- Downregulation of RyhB sRNA which translate into upregulation of genes involved in motility.
- Downregulation of T6SS in chromosome I and II but differential regulation of the T3SS in chromosomes I and II.
- Non-induction of both *tdh*s and VopL, VopT, and VopC when using simple non-conjugated bile salt, and induction of 16 of the T3SS2 genes in absence of VtrB upregulation.
- Increase of cold shock protein CspA and other cold shock protein (VPA0552) by the nutritional upshift at 37C.

Increase in expression of the three CsrB genes was probably due to the faster carbon metabolism required for growing 24 times more rapid at 37 °C. In *E. coli* CsrB RNA functions as an antagonist of CsrA, which is a central component of the carbon storage regulator system and inhibits glycogen biosynthesis, catabolism and gluconeogenesis, among others [19]. Upregulation of CsrB probably increases these processes supporting rapid *Vibrio* growth.

RyhB modulates the expression of several genes that control motility, chemotaxis and biofilm formation, besides iron metabolism*.* In *Vibrio cholerae*, several genes involved in flagellar biosynthesis or chemotaxis are negatively regulated in a *ryhB* mutant*,* and therefore RyhB is necessary for the formation of biofilms and chemotactic motility [26]. By contrast, in *Salmonella Typhimurium*, RyhB2 is involved in the downregulation of the flagellar and chemotaxis genes *flgJ*, *cheY* and *fliF*, thus leading to an increased motility phenotype in a *ryhB2* mutant strain [27]. As observed in our results, there could be an association between the significant downregulation of RhyB and the upregulation of various genes involved in motility under the isolation condition.

All genes differentially expressed for both secretion system type VI(T6SS) were downregulated; 25 of the 29 genes of T6SS1 (VP1386-VP1414) including BfdA, the hemolysin-co-regulated protein (Hcp1), and 17 of the 22 genes of T6SS2, including Hcp2 protein (VPA1025-VPA1046). These results coincide with Salomon et al. in the idea that some of these T6SS play an important role in the adaptability to the environment and fitness of *V. parahaemolyticus* [23], because it is overexpressed in the E condition (12 °C and 3% NaCl). Conversely, there was differential regulation of the T3SS1 and T3SS2, both related to pathogenicity; 31 of the 42 genes that compose the T3SS in chromosome I (VP1656-VP1697) were significantly downregulated (Padj < 1.3E^−6^) while 16 of the 50 genes of T3SS2 (VPA1321-VPA1370) were significantly upregulated (Padj < 1.7E^−7^). This observation is partially in accordance with previous publications where it has been shown that bile salts also induce Vops, the needle-like secretion apparatus, including components such as translocon VopD2, and the expression of *tdh* genes, which belong to pathogenicity island 7 [28–30]. However, unlike these authors, we did not observe some important pathogenicity genes within this pathogenicity island increasing in the presence of bile salts, for example both *tdh* (*tdhA*, VPA1314 and *tdhS*, VPA1378), VPA1370 (VopL), VPA1327 (VopT) and VPA1321 (VopC). This apparent discrepancy could be due to the usage of different bile salts because they have different transcription-inducing activity. We used bile salt from Sigma-Aldrich, which consists of deoxycholate/cholate which according to Gotoh et al. [31] lack or show low transcription induction of *tdh* and Vp-PAI. Taurodeoxycholate and glycodeoxycholate are the bile salt with highest inducing activity. These last two bile salts consist of deoxycholate conjugated with glycine and taurine respectively. In *V. parahaemolyticus*, activation of T3SS2 by bile salts is regulated by VtrA (VPA1332), VtrB (VPA1348) and VtrC (VPA1333). VtrA and VtrC form a functional complex that binds bile salts to activate the cytoplasmic DNA binding domain of VtrA, which in turn induces T3SS2 via the downstream transcription factor VtrB [32]. Since we do not observe a significant upregulation of VtrB (log_2_FC = 0.6), we speculate that upregulation of some genes of T3SS2 could also occur by an independent mechanism. Interestingly, the downregulation of pathogenesis-related genes in condition I (Fig. 2) suggests that besides pathogenesis in humans, these genes are also required for functions that increase *V. parahaemolyticus* fitness in the environment and hence are highly expressed in the condition E. This idea is supported by similar observations of Yang et al. [33] of increased expression of genes responsible for the general secretion pathway, type IV prepilin biogenesis, and pathogenesis when growing at 10 °C instead of 37 °C and by Urmersbach [7] who also observed increased expression of genes related to secretion and pathogenicity comparing growth at 15 °C with 37 °C.

Other mRNAs that changed significantly their expression were the cold shock protein CspA as well as other cold shock proteins (VPA0552). The upregulation of these proteins at 37 °C was surprising and unexpected since other authors has shown that CspA is upregulated in cold shock as expected [33, 34]. However, the observed upregulation of CspA in condition I could be explained by the high nutrients availability in this condition which has been shown increases these proteins [35].

Additionally, the large increase of the five neighbor genes VP1512-VP1516 could be related to the increased growth rate since three of them are related to respiration. The other two are hypothetical proteins that could also participate in this function. Finally, the increase of VP1634 and VP1635, and VP0061 involved in putrescine and spermidine production, commonly associated with putrefaction, may be related to other less known but important functions of these compounds in bacteria when the growth rate is increased, such as rapid growth itself and incorporation into the cell wall [36].

Within 777 genes up- or downregulated in *V. parahaemolyticus*, the most significantly overrepresented categories among upregulated processes were those related to transport and localization, while secretion and pathogenesis were overrepresented among downregulated genes.

Most of differences observed are independent of the availability of nutrients since we used the base medium LB reach in organic nutrients in both conditions. The availability of nutrients in seawater is probably orders of magnitude lower but it is likely that *V. parahaemolyticus* preferentially grow in the ocean when associated to a host supplying nutrients in abundance, specifically when in mollusks or in the intestine of higher animals. Attempts to grow *V. parahaemolyticus* at the low nutrient concentration prevailing in seawater were unsuccessful.

## Conclusions

When *V. parahaemolyticus* is grown in conditions used for laboratory isolation 777 genes are up- or downregulated referred to conditions prevailing in the ocean when organic nutrients are in high supply; the most significantly overrepresented categories among upregulated processes were transport and localization, while secretion and pathogenesis were overrepresented among downregulated genes. Genes with the highest differential expression included the CsrB and RhyB sRNAs and the mRNAs related with secretion, nutritional upshift, respiration and rapid growing.

## Additional file

Additional file 1: RPKM and differential expression values for the *V. parahaemolyticus* genome. Additional file includes ORF and sRNA genes for both condition. (XLSX 395 kb)

## Abbreviations

BGI: Beijing Genomics Institute
BWA: Burrows-Wheeler Aligner
E: Marine-like environmental condition
FC: Fold Change
GO: Gene Ontology
I: Isolation condition
LB: Luria-Bertani
LS: Low protocol sample
ORF: Open reading frame
RPKM: Reads Per Kilobase of transcripted gene per Million mapped reads with features
T3SS: Type III secretion system
T6SS: Type VI secretion system
